# The Basic Premises of EU Regulations Regarding the Safety of Unmanned Aircraft in the Context of their Development Process

**DOI:** 10.1007/s10846-022-01733-x

**Published:** 2022-09-27

**Authors:** Piotr Kasprzyk

**Affiliations:** Institute of Air and Space Law, Lazarski University, Warsaw, Poland

**Keywords:** Unmanned aircraft systems, UAS, Drones, EU regulation

## Abstract

The development of UA is one of the most important challenges for the future of aviation. Consequently, this is one of the major challenges for the future of aviation law, particularly for those legal regulations that aim to provide an adequate level of civil aviation safety. The main goal is to show the results of the analysis of the legal framework created in Europe and to show where Europe is going in the nearest future. The method of study comprised content analysis of existing legislation. Results of the study shows inter alia that although the analysis of the adopted solutions is necessary for a better understanding, a comprehensive assessment of these solutions will be possible at the earliest after the end of the adopted transition periods, i.e. after 2023.

## 1 Introduction

Opening of the aviation market to the civil use of unmanned aircraft (UA) started a *A new era for aviation*. In April 2014 the European Commission published the Communication entitled—*A new era for aviation* (COM (2014) 207 *final)*, that concerned opening of the aviation market to the civil use of unmanned aircraft (UA). [1, 2] The Communication launched a series of actions at EU level, including legislative actions, the Commission pointed out that a number of different unmanned aircraft categories are expected to be operating, diverse in size, performance and type, with some still having a pilot on board, but many remotely piloted or fully automated. The rapid development of drones technology and the necessity of standardization of regulation at the transnational level has become the basis for the development of the European regulation in this scope. [3] The main goal is to show the results of the analysis of the legal framework created in Europe and to show where Europe is going in the nearest future.

It is very important to be aware of the existing regulations. There exist several safety information campaigns but neither information campaigns nor the most perfect regulations will not protect against threats that may be caused by the presence of a drone in a place not intended for. [4–7]

✉ Piotr Kasprzyk
piotr.kasprzyk@lazarski.pl

[1] Institute of Air and Space Law, Lazarski University, Warsaw, Poland

## 2 Regulatory Framework in Europe

When publishing its *Aviation Strategy for Europe* (COM(2015)613 final) in December 2015, the European Commission signalled the need to revise the aviation safety regulatory system. This revision was intended to promote the establishment of more proportionate regulations based on risk assessment. Accordingly, the adoption of a new regulation on common aviation safety regulations was proposed wherein the new regulation would encompass the basic legal framework for the safe development of unmanned aircraft system (UAS) operations in the European Union. The *Strategy* was accompanied by a draft of new rules, and a detailed assessment of the effects of the regulations, including UAS regulations (SWD(2015). The potential approaches were indicated whereby the first option would be to include UAS in the current aviation safety regulations, with the necessary amendments accommodating UAS specificity. The second option would be an approach based on the assessment of the risk posed by a given operation. Appropriate modifications of provisions regarding manned aviation were proposed only for UAS operations with a risk level similar to that of manned aircraft operations, and provisions were made for the certification of equipment and operators, as well as for the licensing of personnel. In the case of the remaining operations, the establishment of new regulations was proposed which would be proportionate to the risk associated with the operations. This second option also provided for a variant where the rules concerning product safety would be used as a regulatory mechanism to ensure a minimal level of safety for small UAS. Ultimately, the latter course was recommended. The works ended with the adoption of *Regulation (EU) 2018/1139 of the European Parliament and of the Council of 4 July 2018 on common rules in the field of civil aviation and establishing a European Union Aviation Safety Agency* (OJ L 212, 22.8.2018) which entered into force on 11 September 2018 [8].

As highlighted in the preamble to *Regulation 2018/1139*, it applies to unmanned aircraft regardless of mass. It was pointed out that due to the diversity of UAS operations, these should be subject to regulations that are proportionate to the risk associated with a particular operation or type of operation. It was therefore decided that for some UAS types, subjecting these to the mechanisms provided for in product safety regulations will be a sufficient way of ensuring safety. It is worth highlighting that Article 55 of *Regulation 2018/1139* introduced a general mechanism to distinguish, in implementing (delegated) acts, between UAS whose production and operation are subject to certification requirements, and the remaining UAS that are subject to more flexible rules. The "certified" UAS category is to be subject to the same essential requirements as any aircraft (airworthiness, crew, operations), which are stipulated in appropriate annexes to the *Basic Regulation*. The remaining UAS categories may be subject to the essential requirements stipulated in Annex IX to this regulation. In other words, two directions for the development of UAS regulations were established. In the case of UAS in the certified category, the regulation provides for the need to include this type of aircraft and their operations in implementing legislation that comprehensively regulates aviation safety. For the remaining types of UAS and their operations, entirely new regulations were established. [8] These were published within less than a year from the adoption of the new *Basic Regulation*, on 11 June 2019, as regulations of the European Commission issued based on Articles 58 and 57 of *Regulation 2018/1139*, respectively:

1. Commission Delegated Regulation (EU) 2019/945 of 12 March 2019 on unmanned aircraft systems and on third-country operators of unmanned aircraft systems (OJ L 152, 11.6.2019) and
2. Commission Implementing Regulation (EU) 2019/947 of 24 May 2019 on the rules and procedures for the operation of unmanned aircraft (OJ L 152, 11.6.2019).
3. Analysis and Assessment of the Provisions of the European Regulations 2019/945 and 2019/947.

These regulations are closely interrelated and although *Delegated Regulation 2019/945* has already entered into force, similarly to the vast majority of the provisions of *Implementing Regulation 2019/947*, it is worth keeping in mind that *Regulation 2019/945* provides for some exceptions during the transition period which is to last until the end of 2023. It must be highlighted that *Implementing Regulation 2019/947* initially assumed a one-year *vacatio legis* and was supposed to be in effect from 1 July 2020 but this date was extended to 31 December 2020 due to the pandemic [9].

It is worth pointing out that a significant part of the detailed solutions, without which both the implementing and delegated regulations would be quite difficult to apply, are included in the guidelines and guidance material that take the form of EASA Executive Director Decisions. The guidelines are called Acceptable Means of Compliance or AMC and constitute non-binding standards. According to EASA, meeting these standards is equivalent to proving compliance with the provisions of the *Implementing Regulation*. Guidance Material or GM, on the other hand, are non-binding examples and explanations that allow to better understand the provisions of the *Implementing Regulation*. The decision 2019/021/R issuing AMC/GM to *Regulation 2019/947* was published on 9 October 2019 (https://www.easa.europa.eu/document-library/agency-decisions/ed-decision-2019021r).

The provisions are quite extensive and detailed. The basic assumptions made during their establishment are laid out in documents from the legislative process involving the implementing regulations, which took place in parallel with the works on the new *Basic Regulation*, including in *'Prototype' Commission Regulation on Unmanned Aircraft Operations* (https://www.easa.europa.eu/system/files/dfu/UAS%20Prototype%20Regulation%20final.pdf and https://www.easa.europa.eu/system/files/dfu/Explanatory%20Note%20for%20the%20UAS%20Prototype%20regulation%20final.pdf), *Notice of Proposed Amendment 2017–05 (A)* and *(B)* (https://www.easa.europa.eu/document-library/notices-of-proposed-amendment/npa-2017-05), and *Opinion 01/2018* (https://www.easa.europa.eu/document-library/opinions/opinion-012018).

First and foremost it must be pointed out that although the analysed provisions mainly involve the safety of UAS use, they are also associated with other issues that are significant for the public acceptance of UAS, such as the protection of privacy and personal data, and environmental protection, including protection from noise. Therefore, the requirements for operator registration or UAS identification are not only aimed at achieving an adequate safety level but are also meant to help solve other issues associated with the public acceptance of UAS.

As far as safety is concerned, the two basic risks associated with UAS operations are air risks and ground risks. Air risks concern collisions with other aircraft, both manned and unmanned. Ground risks, on the other hand, concern collisions with people or elements of critical infrastructure. These risks are different for different categories of UAS operations and may therefore be addressed differently: regulations should be operation centric. Instead of focusing on who is performing a particular operation and why and what aircraft they are using, the focus should be on the operation itself and the risk that it carries. And this risk may materialize in the airspace where the operation is taking place or on the surface of the ground. It is therefore advisable to depart from the traditional approach whereby an aircraft is the starting point for creating safety regulations and concentrate on the operation instead. This will allow the identification and assessment of the risk posed by a given operation. The higher the risk, the higher the requirements aimed at risk mitigation and therefore regulations should be proportionate to the level of risk, i.e. risk based. For example, the mere fact that a commercial operation is performed should not determine whether authorization or operator certification is required. This should depend on the risk involved in a given operation. Moreover, whenever possible, regulations should identify and indicate particular goals or indicators whose achievement is equivalent to ensuring a desired safety level. The way of achieving these should be determined, if possible, by the addressee of the provisions. Performance-based regulations should therefore complement (or even replace) the traditional method of regulating safety requirements by determining increasingly detailed requirements that are to be met by addressees. It should be added that the new rules for the establishment of safety regulations applied in UAS regulations are being promoted in the entire aviation safety regulatory system. Their introduction was part of a revision of the entire system and was reflected in the general part of *Regulation 2018/1139* (*see motive 12 and Article 4 par. 1 and 2*).

Three categories of operations and the associated risks were identified when implementing the above principles.

The **open category** comprises operations that pose no or a very small risk for third parties (on the ground and in the air). Operations in the open category should follow well-defined principles that pilots must be required to know. Moreover, the vast majority of risks should be included in the technical requirements and operational limitations inherent to UAS used in the open category. This is to occur through the use of product compliance mechanisms, resulting in UAS intended for use in the open category having to meet these requirements which will be confirmed by appropriate marking on the product itself (CE mark).

The **specific category** comprises operations that pose a higher risk than operations in the open category, or operations that cannot be performed with the operational limitations for this category. This type of operations will require authorization by an aviation authority (an authority indicated by a member state). Applications for authorization will have to be supported by proof (via risk analysis) that operation safety is ensured at an acceptable level. Such authorization will not be required for operations performed in accordance with a standard scenario, i.e. requirements specified at the regulatory level which result from a risk analysis carried out at the level of the regulator [10].

In the **certified category**, the risks are similar to those for manned aviation. Therefore, solutions may be put in place that are used to ensure manned aviation safety, such as product and operator certification, and the licensing of personnel involved in the operations. [5]

*Regulation 2019/945* regulates in detail the requirements regarding the design and manufacture of UAS intended for use as part of the open category. Instead of the traditional procedures for the certification of equipment, the regulation provides for the use of harmonizing legislation regarding product safety with regard to these UAS. Meanwhile, *Regulation 2019/947* provides detailed regulation of operations performed in the open and specific categories, including the requirements for UAS pilots and operators. Determining the category of the intended UAS operation is therefore essential for the determination of the obligations of UAS pilots and operators.

According to Article 4 of *Regulation 2019/947*, an operation is included in the open category if the following requirements are met. First, the maximum mass of the UAS does not exceed 25 kg and the UAS itself meets the requirements for a given class, as per *Regulation 2019/945*, confirmed by the CE mark. Second, when performing operations, the pilot makes sure that a safe distance from people is kept. Third, the UAS must be kept within line of sight. Fourth, a flight altitude lower than 120 m above terrain (or obstacle passed) must be maintained. Fifth, no items may be dropped off by the UAS and carrying dangerous goods is forbidden. If any one of these requirements is not met, the operation is included in the specific category.

If an operation may not be carried out in accordance with the requirements and limitations specified for the open category, it is included in the specific category, unless the associated risk calls for including it in the certified category. There are three elements, stipulated in *Regulation 2019/947*, that are essential when classifying an operation as pertaining to the specific category:

1. the failure to meet any one of the requirements or limitations stipulated for operations in the open category and a particular subcategory (Article 5 par. 1);

2. the failure to meet the conditions laid out in the provisions as the boundary conditions for the certified category (Article 6 par. 1);
3. risk assessment confirming that there are no grounds to classify a given operation as pertaining to the certified category (Article 6 par. 2).

Three types of operations are included in the certified category. First, operations over assemblies of people defined as situations where the number of persons present prevents them from moving away so as to avoid a potential collision with a UAS over which control has been lost. Second, operations associated with the transport of persons. Third, operations associated with the transport of dangerous goods.

The fundamental principle underlying operations in the specific category is the need to obtain authorization from the appropriate authority in a member state in which the operator is registered (Article 5 par. 1). The application for authorization must be supported by a risk analysis for a given operation (Article 5 par. 2). *Regulation 2019/947* specifies both the requirements regarding the risk analysis itself (Article 11), as well as authorization for the performance of an operation (Article 12). The risk assessment of a given operation which must be performed before applying to the competent authority for its authorization is crucial for operations in the specific category. It is worth pointing out that the provision of Article 11 of *Regulation 2019/947* is specified in great detail in the Acceptable Means of Compliance (AMC) published by EASA. AMC1 to the abovementioned EASA provision recommends the use of the Specific Operations Risk Assessment (SORA) methodology developed by JARUS.

Regulations regarding the specific category and in particular the recommended risk assessment methodology, SORA, have become the subject of further legislative work. Interestingly, proceedings on the amendments to *Regulations 2019/947* and *2019/945* began before these regulations entered into force. EASA published proposed changes to both regulations already in 2019, proposing so-called standard scenarios or STS. These scenarios were meant to enable the performance of operations based on declarations made in advance (instead of required authorization) in the following cases:

1. VLOS operations using UAS with a mass of up to 25 kg, to an altitude of up to 120 m, in areas where people may be present (STS-01),
2. BVLOS operations using UAS with a mass of up to 25 kg, to an altitude of up to 120 m, at a maximum distance of 2 km from the pilot, on the condition that observers are involved (STS-02).

These proposals resulted in the adoption of *Commission Implementing Regulation (EU) 2020/639 of 12 May 2020 amending Implementing Regulation (EU) 2019/947 as regards standard scenarios for operations executed in or beyond the visual line of sight (OJ L 150, 13.5.2020)* and *Commission Delegated Regulation (EU) 2020/1058 of 27 April 2020 amending Delegated Regulation (EU) 2019/945 as regards the introduction of two new unmanned aircraft systems classes (OJ L 232, 20.7.2020).* The provisions were supposed to enter into force on 2 December 2021 but it became clear that the so-called harmonized standards for UAS requirements would not be ready by that date. Therefore, another implementing regulation was issued to postpone the starting date of the application of standard scenarios STS-01 and STS-02 (Commission Implementing Regulation (EU) 2021/1166 of 15 July 2021 *amending Implementing Regulation (EU) 2019/947 as regards postponing the date of application for standard scenarios for operations executed in or beyond the visual line of sight*, OJ L 253, 16.7.2021).

## 3 *Quo Vadis* Europe?

In 2020–2021 quite significant amendments and supplementations were also made in EASA guidelines, i.e. the AMC/GM to *Regulation 2019/947* which were first published in October 2019 as *EASA Executive Director Decision 2019/021/R*. These were the following, respectively:

1. *Executive Director Decision 2020/022/R* of 15.12.2020 (https://www.easa.europa.eu/document-library/agency-decisions/ed-decision-2020022r) pursuant to which the requirements were amended for UAS use in the specific category and the principles of the interoperability of UAS registration systems were determined,
2. *Executive Director Decision 2022/002/R* of 07.02.2022 (https://www.easa.europa.eu/document-library/agency-decisions/ed-decision-2022002r) pursuant to which e.g. the digital standard was determined for the establishment of so-called geographical zones and the provisions of the specific category were further clarified (obtaining operational authorization, pilot training, risk assessment for certain BVLOS operations).

Changes introduced in 2020 to the AMC regarding the SORA methodology (AMC to Article 11 of *Regulation 2019/947*) deserve particular attention. As a result of these changes, the requirements for determining the ground risk class were raised. Consequently, UAS intended for use in particular operations in the specific category (BVLOS over inhabited areas, mass exceeding 4 kg) will, as a general rule, have to be certified UAS. The changes were proposed in

NPA 2020–07 (https://www.easa.europa.eu/document-library/notices-of-proposed-amendment/npa-2020-07) and make reference to an incident that occurred in May 2019 in which the emergency system was activated and a parachute opened during a drone flight (a test flight for Swiss Post), however, due to a malfunction of the rescue system, the drone collided with the ground near a kindergarten backyard.

The change in SORA methodology mentioned above is associated with a solution adopted in Article 40 par. 1 letter 4 of *Regulation 2019/945*. According to this provision, there is an obligation for UAS performing operations in the specific category to be certified in an attempt to mitigate risk to an acceptable level, wherein this obligation is determined via risk analysis.

The process of UAS certification by EASA requires the establishment of certification requirements that will form the basis for aircraft certification as per the provisions of *Commission Regulation (EU) 748/2012 of 3 August 2012 laying down implementing rules for the airworthiness and environmental certification of aircraft (OJ 224, 21.08.2021).* For now, these requirements are expected to be established in 2023–2024 (CS-UAS, CS-Light UAS). The lack of these requirements renders the certification process impossible. Under certain circumstances, however, *Regulation (EU) 748/2012* does envision aviation equipment certification based on so-called special conditions. In July 2020, EASA published a draft version of special conditions for UAS used in operations in the specific category in cases where UAS certification is necessary based on risk analysis (https://www.easa.europa.eu/document-library/product-certification-consultations/proposed-special-condition-light-uas). However, an a priori limit of 600 kg MTOM was set. The special conditions [11] were ultimately published separately for UAS that pose a medium risk in operations in the specific category (December 2020) and for UAS that pose a high risk in the specific category (December 2021).

The special conditions deal with the structure, design and constructions of particular UAS elements, drive units, systems, control stations, as well as the UAS communication and navigation system. The main assumption for the special conditions is a departure from the traditional method of specifying requirements in a detailed way and instead identifying a safety goal. This goal is defined e.g. by referring to the probability factor of a fatal accident involving a third party on the ground. It therefore requires data such as the population density in the area over which the operations will take place, the technical data (damage causing potential) of a given UAS, or the number of hours that a particular UAS model has performed without malfunction or dangerous situation. The latter refers to events caused by technical issues that are associated with the design of the aircraft and not accidents due to other causes (improper handling, piloting error, atmospheric conditions, collision). In other words, the special conditions focus on the need to indicate safety goals (infallibility of the construction) rather than on determining detailed requirements concerning the construction itself. This performance-based approach has been used for some time in certain areas of "traditional" manned aviation (e.g. general aviation planes). In fact, both EASA (EASA SC-RPAS.1309) and JARUS (JARUS AMC. RPAS.1309) recommended (and adopted) this approach in earlier documents concerning UAS airworthiness. As highlighted in literature, this approach seems to be the most appropriate for the development of unmanned aviation.

However, it must be pointed out that UAS certification is required as a condition for performing operations in the specific category in cases where the operation is assessed as high risk according to SORA methodology. If the risk is assessed as medium, an adequate level of safety is ensured through verification of the UAS design process by EASA. The obligation to perform such verification (and the determination of its scope) may result from the risk assessment performed by the operator or be the condition on which the competent authority issues authorization. According to Article 77 of *Basic Regulation 1138/2018*, however, the verification itself of the UAS design falls within the exclusive competence of EASA. An EASA document issued in March 2021 is dedicated to UAS design verification for operations in the specific category (https://www.easa.europa.eu/newsroom-and-events/press-releases/easa-issues-guidelines-design-verification-drones-operated). There are also plans to publish further amendments to the AMC to *Regulation 2019/947*. It must be highlighted, however, that EASA issued its first design verification report for a UAS intended for use in the specific category in July 2021; the UAS was called Volocopter (https://www.easa.europa.eu/newsroom-and-events/press-releases/easa-issues-first-approval-defined-drone-operations-volocopter).

As far as operations in the certified category are concerned, no draft implementing regulations (or amendments to implementing regulations) have been published so far, and only the commencement of works on certification requirements and provisions for particular groups of operations were announced:

1. UAS that will have the capacity to operate from airports, in controlled airspace, according to IFR regulations, performing cargo flights
2. UAS that will take off/land in urban environments and use designated air corridors in which U-Space services are provided.

## 4 Conclusions

The process of establishing and developing EU regulations regarding the safety of UAS operations highlighted in this paper leads to the following conclusions. It is too early to make a comprehensive assessment of these regulations. Despite the fact that a certain framework for the system was established in *Basic Regulation 1138/2018*, crucial solutions are decided on at the level of delegated and implementing regulations. The adopted pace of works on these regulations, however, causes provisions to be passed in the scope that is ready at a given moment, with amendments or new solutions (such as standard scenarios) being introduced later on. Moreover, quite relevant regulatory solutions are left behind to become EASA guidelines to these regulations. For example, the determination of the types of UAS operations in the specific category that require certification results from the risk assessment methodology (SORA) adopted in the guidelines. Additionally, EASA guidelines undergo quite extensive revisions as well.

Consequently, although analysing the adopted solutions is necessary to gain a better understanding of these, a comprehensive assessment will only be possible once the adopted transition periods end, i.e. after 2023. That is assuming that in 2022–2023 the process of completing the implementing regulations on UAS in the open and certified categories is finalized, similarly to the process of further clarifying these regulations in EASA guidelines. Moreover, the first concrete draft provisions for operations in the certified category, especially draft certification guidelines must also be established. This, too, is scheduled for 2023 at the earliest.

This does not, however, affect the positive assessment of the adopted approach to establish new solutions that are adjusted to the specificity and risk involved in new technology. An alternative approach whereby solutions developed for manned aviation are being adapted to unmanned aviation seems justified only to a limited extent. The division of operations into operations in the open and specific categories on the one hand and in the certified category on the other hand narrows this alternative down to the certified category.

**Author Contributions** This article is not a multi-author publication.

**Funding** Author declare that no funds, grants, or other support were received during the preparation of this manuscript.

**Code Availability** N/A.

## Declarations

**Ethics Approval** N/A

**Consent to Participate** N/A

**Consent to Publish** N/A

**Competing Interests** Author have no relevant financial or non-financial interests to disclose.

**Open Access** This article is licensed under a Creative Commons Attribution 4.0 International License, which permits use, sharing, adaptation, distribution and reproduction in any medium or format, as long as you give appropriate credit to the original author(s) and the source, provide a link to the Creative Commons licence, and indicate if changes were made. The images or other third party material in this article are included in the article's Creative Commons licence, unless indicated otherwise in a credit line to the material. If material is not included in the article's Creative Commons licence and your intended use is not permitted by statutory regulation or exceeds the permitted use, you will need to obtain permission directly from the copyright holder. To view a copy of this licence, visit http://creativecommons.org/licenses/by/4.0/.

## Data Availability

Data sharing not applicable to this article as no datasets were generated or analysed during the current study.

## Code Availability

N/A.
